# The Protective Effects of Annexin A1 in Acute Lung Injury Mediated by Nrf2

**DOI:** 10.1002/iid3.70111

**Published:** 2025-01-14

**Authors:** Hui Huang, Yuqin Shi, Yuequan Zhou

**Affiliations:** ^1^ Department of Stomatology Liyuan Hospital, Tongji Medical College, Huazhong University of Science and Technology Wuhan Hubei China; ^2^ Department of Respiratory and Critical Care Medicine Liyuan Hospital, Tongji Medical College, Huazhong University of Science and Technology Wuhan Hubei China

**Keywords:** acute lung injury, Annexin A1, lipopolysaccharide, NF‐κB, Nrf2

## Abstract

**Background:**

Acute lung injury (ALI), one of the most severe respiratory system diseases, is prevalent worldwide. Annexin A1 (AnxA1) is an important member of the annexin superfamily, known for its wide range of physiological functions. However, its potential protective effect against lipopolysaccharide (LPS)‐induced ALI remains unclear.

**Materials and Methods:**

Mice were divided into four groups: Sham, LPS + vehicle, LPS + 0.1 μg AnxA1, and LPS + 0.5 μg AnxA1. Lung injury was assessed through histopathology, pulmonary wet‐to‐dry (W/D) ratio, cell counting of bronchoalveolar lavage fluid (BALF), oxidative stress analysis, and noninvasive pulmonary function testing. Gene and protein expression levels were measured using RT‐PCR, ELISA, and western blot analysis.

**Results:**

AnxA1 alleviated LPS‐induced ALI by protecting lung tissue from damage, reducing the lung wet/dry (W/D) weight ratio, and improving LPS‐induced impaired lung function. Interestingly, administration of AnxA1 was found to repress the infiltration of inflammatory cells by decreasing the total cell count, neutrophils, and protein concentrations in bronchoalveolar lavage fluid (BALF). AnxA1 mitigated the inflammatory response in the pulmonary tissue by lowering the levels of IL‐1β, IL‐6, and TNF‐α in BALF of ALI mice. Additionally, AnxA1 attenuated oxidative stress in lung tissues of ALI mice by restoring the activity of catalase (CAT), SOD, and glutathione (GSH) but reducing the levels of malondialdehyde (MDA). We also found that AnxA1 suppressed activation of the NLRP3 signaling pathway. Mechanistically, AnxA1 activated the Nrf2/HO‐1 signaling pathway while preventing the activation of NF‐κB.

**Conclusion:**

Collectively, these findings suggest that AnxA1 alleviates LPS‐induced ALI and might be a promising novel therapeutic agent against LPS‐induced ALI.

## Graphical abstract

Annexin A1 improves LPS‐induced impaired lung function and alleviates pathological changes in the lung tissues of ALI mice. Annexin A1 ameliorates inflammation and oxidative stress in the BALF of ALI mice. The protective effects of Annexin A1 are mediated by the Nrf2/HO‐1/NF‐κB pathway.

## 1 Introduction

Acute lung injury (ALI) is an acute hypoxic respiratory failure or dysfunction caused by a series of factors during non‐cardiogenic diseases such as severe infections, burns, shock, etc. It is the most common cause and presentation of acute respiratory failure. ALI is characterized by decreased lung compliance, reduced volume, and severe ventilation/perfusion (V/Q) mismatch as pathophysiological features. The clinical symptoms include progressive and irreversible hypoxemia and respiratory distress. The imaging examination of the lung often reveals exudative lesions and nonuniform manifestations [1, 2]. Epidemiological investigations have shown that ALI is a common critical illness, with multiple risk factors contributing to its development. Studies have found that the incidence of ALI can be as high as 25%–50% in cases of severe infection and up to 11%–25% when multiple injuries occur [3, 4]. Acute respiratory distress syndrome (ARDS) is the result ALI progression, and the mortality rate can be as high as 50% [5], which poses a great threat to the lives of critically ill patients and significantly affects the quality of life of surviving patients. Currently, the pathogenesis of ALI remains unclear, and treatment options are limited. Exploring its unknown pathogenesis and finding new ideas for the clinical treatment of ALI/ARDS has significant clinical value. LPS plays a vital mediating role in the pathogenesis of ALI. LPS activates inflammatory cells in the lung to release inflammatory factors, leading to lung tissue inflammation and alveolar wall damage. The release of inflammatory factors causes vascular leakage and pulmonary edema, severely damaging lung function. Additionally, LPS‐induced inflammatory reactions may further aggravate lung injury by activating the coagulation system and promoting platelet aggregation [5, 6]. On the other hand, LPS‐induced oxidative stress (OS) is also involved in promoting the development of ALI [7]. As an important component of the cell wall of Gram‐negative bacteria, LPS stimulation leads to excessive production of reactive oxygen species (ROS) and reactive nitrogen species (RNS). Most of these oxidants originate from innate immune cells (especially macrophages and recruited neutrophils) within damaged lung tissue, further exacerbating lung inflammation and tissue damage [8, 9]. Regulating LPS‐induced inflammatory reactions and OS is an important strategy for treating ALI.

Annexin A1 (AnxA1) is a highly conserved membrane protein that is widely present in various tissues and organs of the human body, including vascular endothelial cells, the liver, kidneys, lungs, intestines, and testes [10]. AnxA1's primary function is to bind and internalize high‐density lipoprotein (HDL) on the cell membrane, transporting cholesterol and phosphatidylcholine to the liver and kidneys for metabolism and excretion. This process helps maintain the balance of cholesterol levels in the body [11, 12]. AnxA1 is also considered an important receptor involved in various pathophysiological processes, such as infections, tumors, inflammation, and cardiovascular diseases [13]. Furthermore, research has shown that AnxA1 is engaged in processes such as cholesterol efflux, cell apoptosis, oxidative stress, immune regulation, and neuroprotection, demonstrating its multifunctionality [14]. Herein, the potential clinical value of AnxA1 in regulating inflammation and OS and treating ALI was evaluated.

## 2 Materials and Methods

### 2.1 Animals and Treatments

This study was approved by the Ethics Committee of Liyuan Hospital, Tongji Medical College, Huazhong University of Science and Technology (Approval #21‐AEA0032). Forty BALB/c mice were divided into four groups: Sham, LPS+ vehicle, LPS + 0.1 μg AnxA1, and LPS + 0.5 μg AnxA1. In the LPS+ vehicle group, mice were intranasal administered with 50 μL LPS (#L6529; Sigma‐Aldrich, USA) [15] 0 μL of blank vehicle. In the Sham group, BALB/c mice were intranasally administered with 100 μL of normal saline. In the LPS + 0.1 μg AnxA1 and LPS + 0.5 μg AnxA1 groups, BALB/c mice were intranasally administered with 50 μL of LPS and 50 μL of vehicle containing 0.01 and 0.5 μg of AnxA1 (#ab86446, Abcam, USA), respectively, at a 72‐h time point.

### 2.2 Histopathology and Assessment of Lung Injury Score

Lung tissue was fixed in 10% formalin and embedded in paraffin. Sections of 5 μm were sliced with a microtome (RM2255, Leica, Germany). For the rehydration process, lung tissue specimens were placed in xylene solution to remove paraffin, with each step lasting for 15 min. The slices were then sequentially immersed in ethanol at concentrations of 100%, 95%, 85%, and 75% for 5 min each, followed by a rinse in distilled water for 5 min. The slides were stained with Mayer's hematoxylin staining solution (#41325, Sigma‐Aldrich, USA) for 1–5 min, then rinsed with distilled water and returned to blue using a PBS washing solution. Next, the tissue was stained with Eosin staining reagent (#63184, Sigma‐Aldrich, USA) for 5 s. The slides were then thoroughly rinsed with distilled water to remove excess Eosin staining. The lung tissue slices were dehydrated again using a gradient of alcohol (95%–100%) for 5 min at each level. The dehydrated lung tissue slices were then placed in xylene solution for 10 min, repeated twice. ProLong gold antifade mounting media (# P10144, Thermo Scientific, Germany) was used for mounting the slides. Lung tissue pathological changes were observed under a microscope (Axio Imager 2, Zeiss, Germany), followed by scoring lung tissue injury according to a semi‐quantitative scoring standard based on pathological changes in the lung tissue. Scoring criteria included assessment of congestion, hemorrhage, neutrophil infiltration or aggregation in alveoli/vascular walls, and alveolar wall thickening and/or membrane formation. Scoring was conducted as follows: no or minimal changes were scored as 0, mild changes as 1, moderate changes as 2, severe changes as 3, and very severe changes as 4 [16].

### 2.3 Measurement of Pulmonary W/D Ratio

The lung wet‐to‐dry (W/D) ratio was measured as an indicator of pulmonary edema. Briefly, the pulmonary tissue was excised, and the tissue was immediately wrapped in clean aluminum foil. The wet weight was accurately measured and recorded as W. The tissue was then dried in a constant temperature dry‐air incubator (BB‐BOV‐D35, Biobase, China) at 60°C for 48 h, and the dry weight was measured and recorded as D. The W/D ratios of the lung tissues were determined based on these recorded values [17].

### 2.4 Collection of Bronchoalveolar Lavage Fluid (BALF) and Cell Counting

Mice were anesthetized using sterile continuous 2% Isoflurane gas spray anesthesia and then fixed on their backs on a surgical platform. The chest cavity and trachea were opened along the midline using sterile surgical scissors and forceps. A sterile tube was inserted to instill and withdraw sterile saline three times, with 1 mL each time. The collected saline was placed in a sterile centrifuge tube and centrifuged at 1250 g for 10 min. The supernatant was collected as BALF. The BALF was thoroughly mixed, and 10 μL of the suspension was added to an automatic cell counter plate. The plate was then inserted into a cell counter (#PHCC360KIT, Millipore, USA), and the relevant parameters were adjusted to obtain the total cell count and the count of neutrophils. A bicinchoninic acid (BCA) kit (71285‐M, Merck, USA) was used for determining the protein contents in BALF.

### 2.5 Measurement of Lung Function

Twenty‐four hours post‐dosing, the peak expiratory flow (PEF), airway resistance (RAW), and changes in dynamic lung compliance (Cdyn) of each mouse were measured using the noninvasive pulmonary function testing system (#NAM, DSI BUXCO, USA) to assess their lung function.

### 2.6 Enzyme Linked Immunosorbent Assay (ELISA)

BALF was collected from each animal. The levels of pro‐inflammatory cytokines in the BALF were determined using commercial kits (Abcam, USA) according to the manufacturer's instructions: IL‐1β (#ab197742, Abcam, USA), IL‐6 (#ab222503, Abcam, USA), and TNF‐α (#ab208348, Abcam, USA). The BALF supernatant, collected by centrifugation, was mixed with samples and loaded into the corresponding wells of a reaction plate. The plate was then incubated at 37°C for 90 min, after which the liquid in the plate was discarded. Subsequently, 100 μL of biotin‐labeled antibody was added to each well (except for the blank well), and the plate was incubated at 37°C for 60 min, followed by five washes with washing buffer. Then, 100 μL of ABC working solution was added (except for the blank well), and the plate was incubated at 37°C for 30 min. After incubation, 90 μL of chromogenic agent was added, and the plate was agitated and placed in a 37°C environment for 15 min. Finally, 100 μL of termination agent was added, and the absorbance at 450 nm was measured. The concentration was calculated by fitting the standard curve.

### 2.7 Assessment of Os Parameters

Catalase (CAT) activity in BALF was assessed using the ultraviolet spectroscopy method with a commercial kit (#ab118184, Abcam, USA). Malondialdehyde (MDA) levels were determined using the TBARS method with a commercial kit (#BAQ068, G‐Biosciences, USA). Superoxide dismutase (SOD) activity in BALF was analyzed using the WST‐1 method with a commercial kit (#BC5165, Solarbio, China). Glutathione peroxidase (GSH) activity was measured using a commercial kit (#ab112132, Abcam, USA). All procedures were performed according to the manufacturers’ instructions.

### 2.8 RT‐PCR Assay

According to the instructions of the total RNA extraction kit (#AM1830, Thermo Scientific, Germany), RNA was isolated from mouse lung tissue, and its concentration was determined using a microvolume nucleic acid protein concentration meter (DeNovix DS‐11, Thermo Scientific, Germany). Once confirmed to be free of contamination, the total RNA was reverse‐transcribed into cDNA using a 20 μL reaction system, following the instructions of the reverse transcription kit (#4368814, Thermo Scientific, Germany). Primers, cDNA, fluorescent dye, and DEPC water were mixed in a 10 μL reaction system and placed in a high‐throughput real‐time fluorescence quantitative PCR instrument (BD, USA) for detection. Finally, the relative expression levels of each gene were calculated using the 2^‐ΔΔCt^ method.

### 2.9 Western Blot Analysis Assay

The mouse lung tissue was homogenized in RIPA buffer (#89901, Thermo Scientific, Germany), and the protein contents in the homogenate were determined using a BCA kit (71285‐M, Merk, USA). Subsequently, 6× loading buffer was added to the homogenate, and the specimen was applied onto an SDS‐PAGE gel for separation. After separation, the proteins were transferred onto a membrane and left for 2 h. The membrane was then blocked with 5% BSA for 2 h and subsequently incubated with the corresponding primary antibody against NLRP3 (1:2000, #15101, Cell Signaling Technology, USA), Caspase‐1 (1:1000, #83383, Cell Signaling Technology, USA), GSDMD N (1:1000, #ab215203, Abcam, USA), GSDMD FL (1:2000, #ab219800, Abcam, USA), Nrf2 (1:2000, #12721, Cell Signaling Technology, USA), HO‐1 (1:2000, #70081, Cell Signaling Technology, USA), p‐IκBα (1:500, #2859, Cell Signaling Technology, USA), p‐NF‐κB p65 (1:500, #3033, Cell Signaling Technology, USA), and β‐actin (1:5000, #4967, Cell Signaling Technology, USA) overnight at 4°C. After washing with TBST buffer, the membrane was incubated with a secondary antibody (1:2000, #7074, Cell Signaling Technology, USA) for 1 h and then exposed. The obtained bands were analyzed using Gel‐Pro Analyzer 4.0 (Media Cybernetics, L.P.).

The expression levels of NLRP3, Caspase‐1, Nrf2, and HO‐1 were normalized to β‐actin. Similarly, the expression levels of GSDMD‐N, p‐IκBα, and p‐NF‐κB p65 were normalized to GSDMD‐FL, IκBα, and NF‐κB p65, respectively.

### 2.10 Statistical Analysis

All results are described as the means ± SEM, and GraphPad Prism 8 was used in the generation of graphs. The statistical significance of the data was analyzed by ANOVA and Tukey's Post hoc test in SPSS 23.0. A *p*‐value below 0.05 was identified as statistically significant.

## 3 Results

### 3.1 AnxA1 Alleviates Pathological Changes in Lung Tissues of ALI Mice

**Figure 1:**
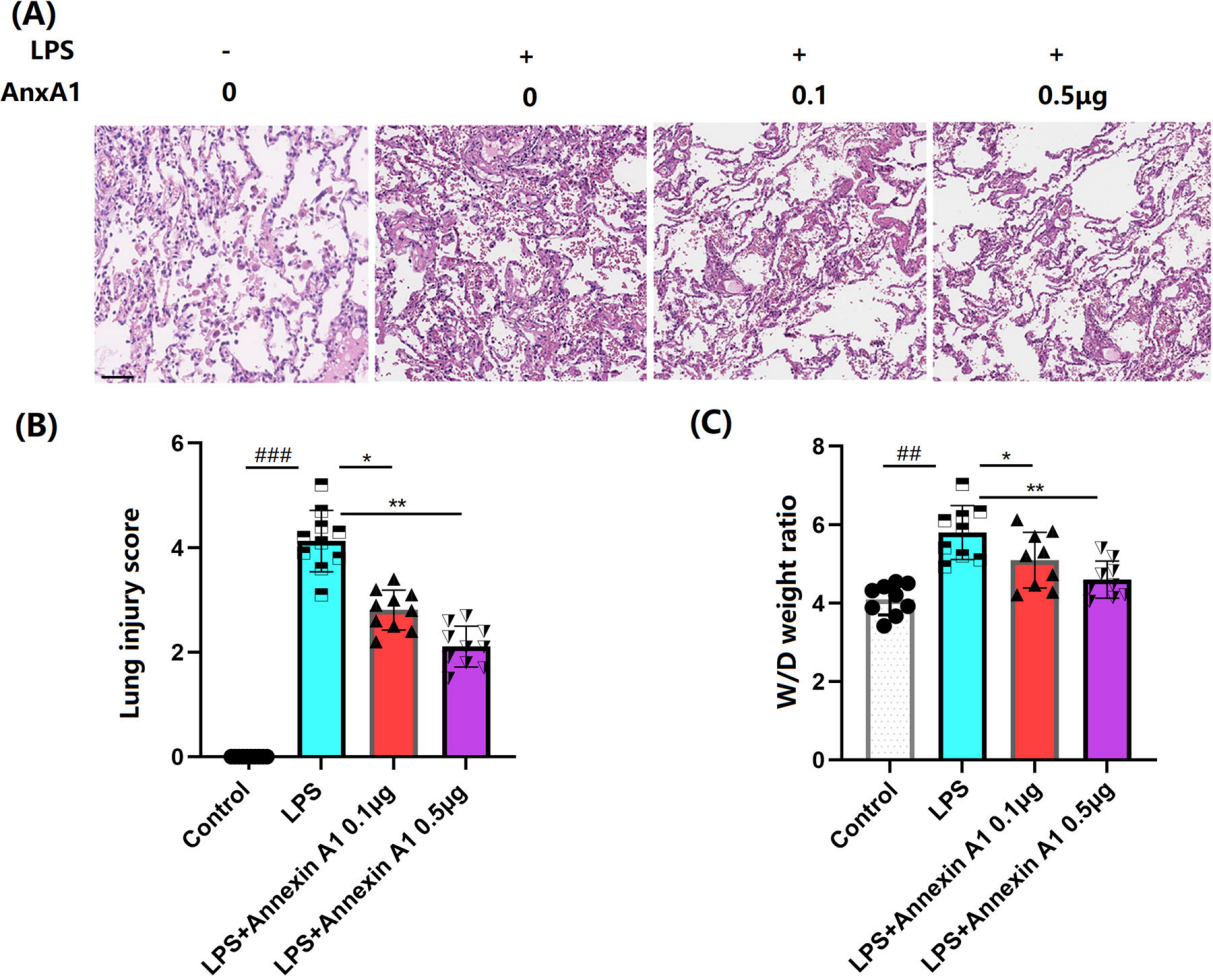
Annexin A1 alleviated pathological changes in lung tissues of acute lung injury (ALI) mice. (A) Representative hematoxylin and eosin (HE) staining images of the mice lung tissues; Scale bars, 100 μm; (B) Lung injury score; (C) Lung wet weight/dry (W/D) weight ratio (##, ###, *p* < 0.01, 0.005 vs. vehicle group; *, **, *p* < 0.05, 0.01 vs. ALI group, N = 9–10, two‐way ANOVA).

Pathological changes in lung tissues are illustrated in Figure 1A. No abnormalities were observed in the lung tissue of the Sham group mice. The lung tissue of mice in the LPS+ vehicle group showed significant infiltration of inflammatory cells and bleeding, as well as thickening of the alveolar walls and narrowing of the alveolar spaces. The pathological condition was remarkably improved by 0.1 and 0.5 μg AnxA1. The lung injury score was strikingly boosted from 0 to 4.1 in ALI mice, which was sharply restrained to 2.8 and 2.1 by 0.1 and 0.5 μg AnxA1, respectively (Figure 1B). W/D ratios in the Sham, LPS+ vehicle, LPS + 0.1 μg AnxA1, and LPS + 0.5 μg AnxA1 groups were 4.1, 5.8, 5.1, and 4.6, respectively (Figure 1C).

### 3.2 AnxA1 Improves LPS‐Induced Impairment of Lung Function

**Figure 2:**
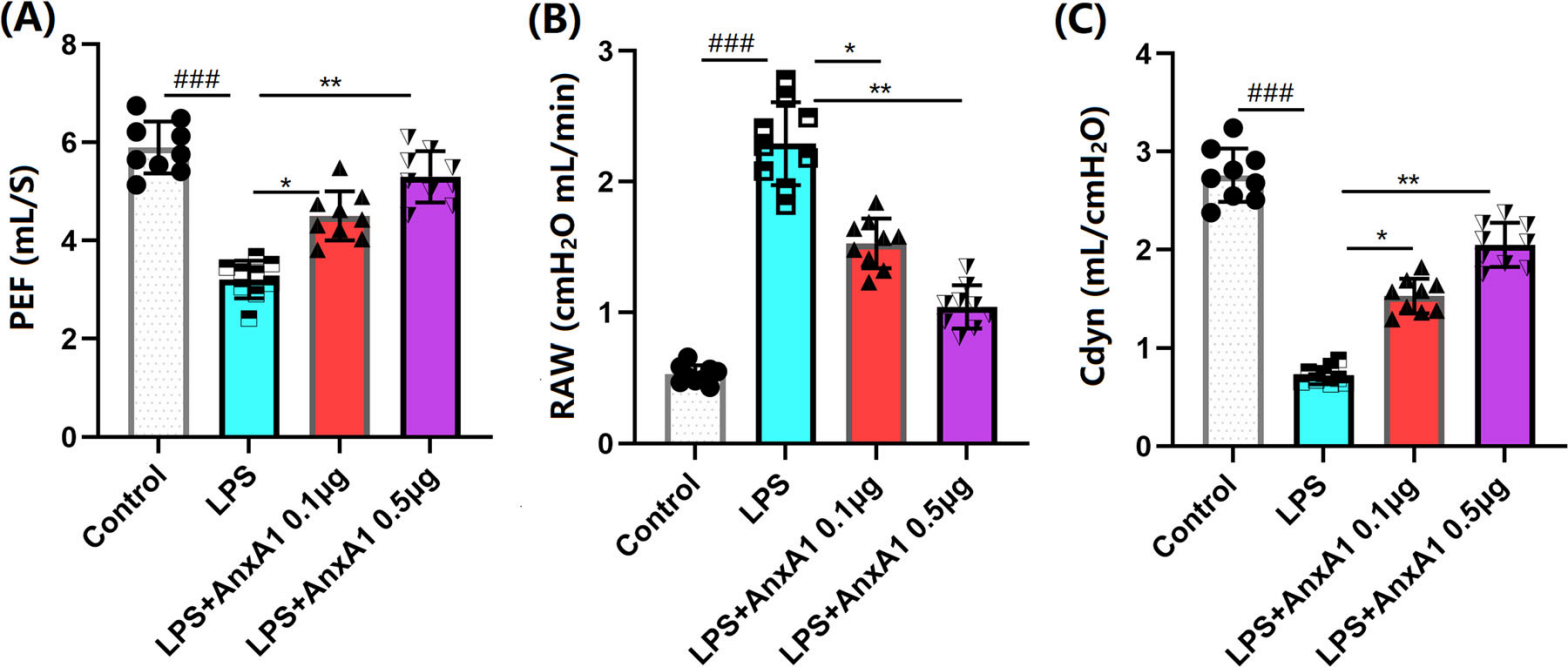
Annexin A1 improved lung function impaired by LPS‐induced injury. (A) PEF; (B) RAW; (C) Cdyn (###, *p* < 0.005 vs. vehicle group; *, **, *p* < 0.05, 0.01 vs. ALI group, *N* = 9, two‐way ANOVA).

Subsequently, the lung function of each animal was assessed. PEF values in ALI mice declined from 5.9 to 3.2 mL/s, which sharply increased to 4.5 and 5.3 mL/s by 0.1 and 0.5 μg AnxA1, respectively (Figure 2A). RAW values in the Sham, LPS+ vehicle, LPS + 0.1 μg AnxA1, and LPS + 0.5 μg AnxA1 groups were 0.53, 2.29, 1.53, and 1.04 cmH_2_O mL/min, respectively (Figure 2B). In addition, in ALI mice, the Cdyn value was restrained from 2.76 to 0.72 mL/cmH_2_O, which was strikingly boosted to 1.53 and 2.05 mL/cmH_2_O by 0.1 and 0.5 μg AnxA1, respectively (Figure 2C).

### 3.3 AnxA1 Inhibits the BALF Inflammatory Environment in ALI Mice

**Figure 3:**
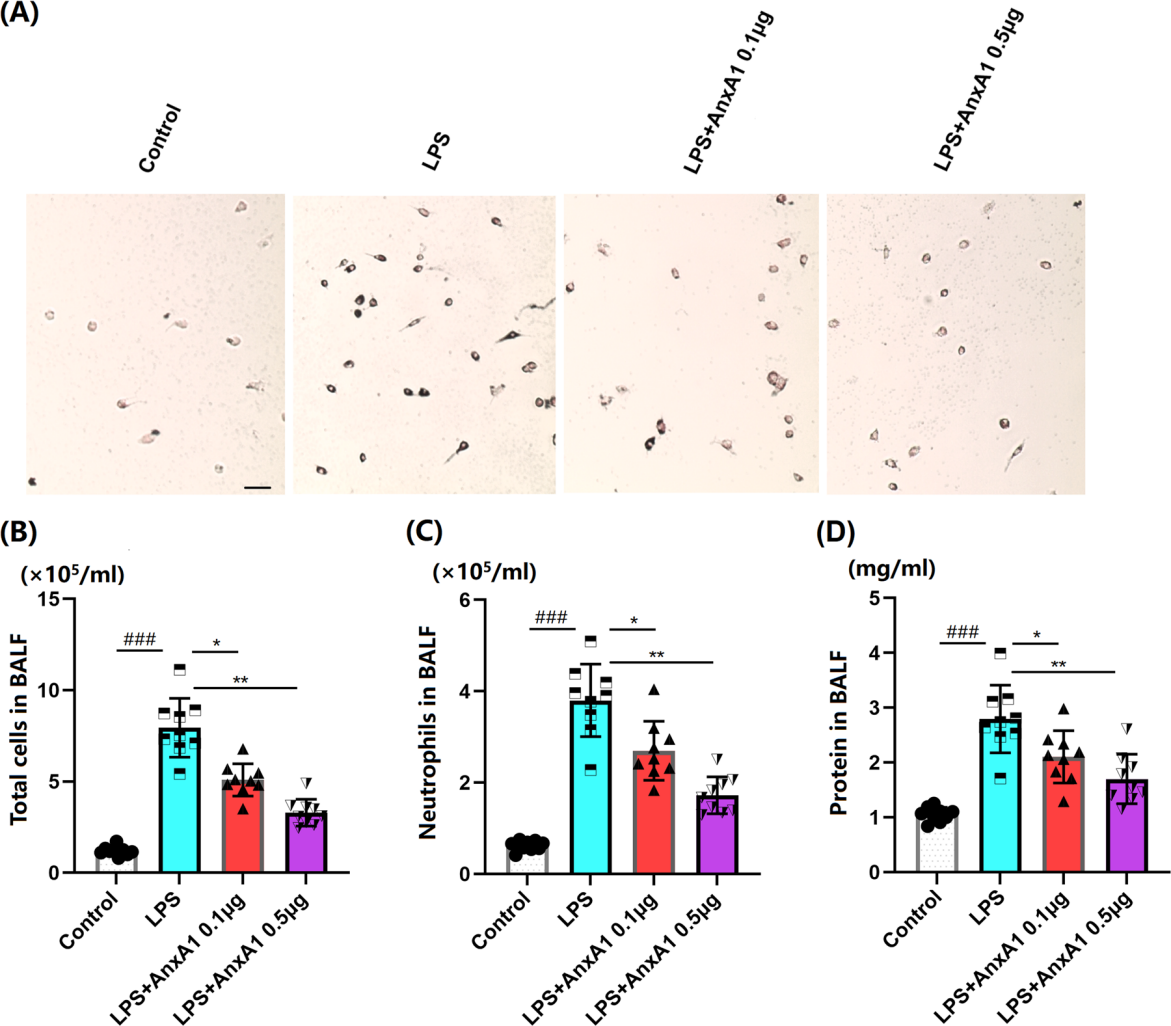
Annexin A1 repressed the infiltration of inflammatory cells and protein in the BALF of ALI mice. (A) Representative images of total cells and neutrophils in BALF; Scale bar, 50 μm; (B) Total cell count in BAL; (C) Neutrophil count in BALF; (D) Protein concentration in BALF (###, *p* < 0.005 vs. vehicle group; *, **, *p* < 0.05, 0.01 vs. ALI group, *N* = 9, two‐way ANOVA).

Subsequently, the impact of AnxA1 on inflammatory cell infiltration was assessed. As illustrated in Figure 3A, LPS stimulation led to a substantial rise in both total cellularity and neutrophil counts in the BALF. However, this increase was mitigated in a dose‐dependent fashion following AnxA1 administration, as depicted in Figure 3B,C. Correspondingly, protein concentration in BALF in ALI mice was markedly boosted from 1.05 to 2.8 mg/mL, which was visibly decreased to 2.1 and 1.7 mg/mL by 0.1 and 0.5 μg AnxA1, respectively (Figure 3D).

### 3.4 AnxA1 Ameliorates BALF Inflammation in ALI Mice

**Figure 4:**
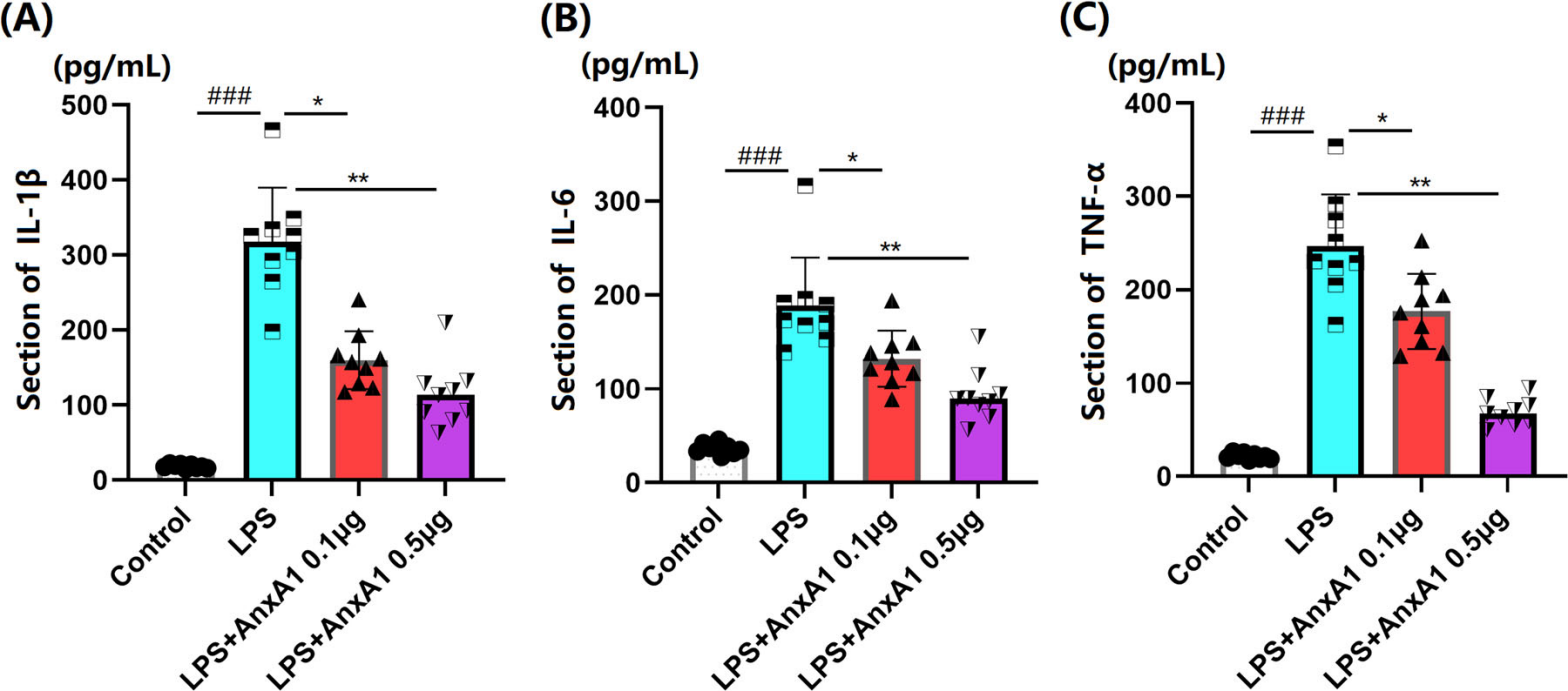
Annexin A1 ameliorated inflammation in the BALF of ALI mice. The concentration of (A) IL‐1β; (B) IL‐6; and (C) TNF‐α in the BALF (###, *p* < 0.005 vs. vehicle group; *, **, *p* < 0.05, 0.01 vs. ALI group, *N* = 9, two‐way ANOVA).

IL‐1β contents in the BALF of ALI mice were enhanced from 18.6 to 316.7 pg/mL, which were sharply restrained to 159.7 and 114.8 pg/mL by 0.1 and 0.5 μg AnxA1, respectively (Figure 4A). IL‐6 levels in the Sham, LPS+ vehicle, LPS + 0.1 μg AnxA1, and LPS + 0.5 μg AnxA1 groups were 36.9, 188.4, 132.2, and 93.5 pg/mL, respectively (Figure 4B). Furthermore, TNF‐α contents in ALI mice were enhanced from 22.6 to 246.7 pg/mL, which were markedly restrained to 176.6 and 69.4 pg/mL by 0.1 and 0.5 μg AnxA1, respectively (Figure 4C).

### 3.5 AnxA1 Represses Os in ALI Mice

**Figure 5:**
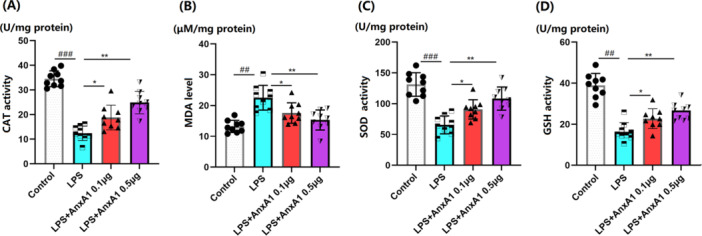
Annexin A1 repressed oxidative stress in the lung tissues of acute lung injury (ALI) mice. (A) Catalase (CAT) activity; (B) Malondialdehyde (MDA) level; (C) Superoxide dismutase (SOD) activity; (D) Glutathione peroxidase (GSH) activity (##, ###, *p* < 0.01, 0.005 vs. vehicle group; *, **, *p* < 0.05, 0.01 vs. ALI group, *N* = 9, two‐way ANOVA).

In ALI mice, the CAT activity in lung tissues was significantly declined from 34.5 to 12.4 U/mg protein, which was notably boosted to 18.8 and 24.9 U/mg protein by 0.1 and 0.5 μg AnxA1, respectively (Figure 5A). Furthermore, MDA contents in the Sham, LPS+ vehicle, LPS + 0.1 μg AnxA1, and LPS + 0.5 μg AnxA1 groups were 13.2, 21.4, 17.6, and 15.3 μM/mg protein, respectively (Figure 5B). SOD activities in ALI mice were markedly repressed from 131.2 to 65.6 U/mg protein, which were considerably enhanced to 89.4 and 108.3 U/mg by 0.1 and 0.5 μg AnxA1, respectively (Figure 5C). Additionally, the GSH activity in the Sham, LPS+ vehicle, LPS + 0.1 μg AnxA1, and LPS + 0.5 μg AnxA1 groups was 38.9, 16.3, 22.7, and 26.1 U/mg protein, respectively (Figure 5D).

### 3.6 AnxA1 Suppresses NLRP3 Activation in ALI Mice

**Figure 6:**
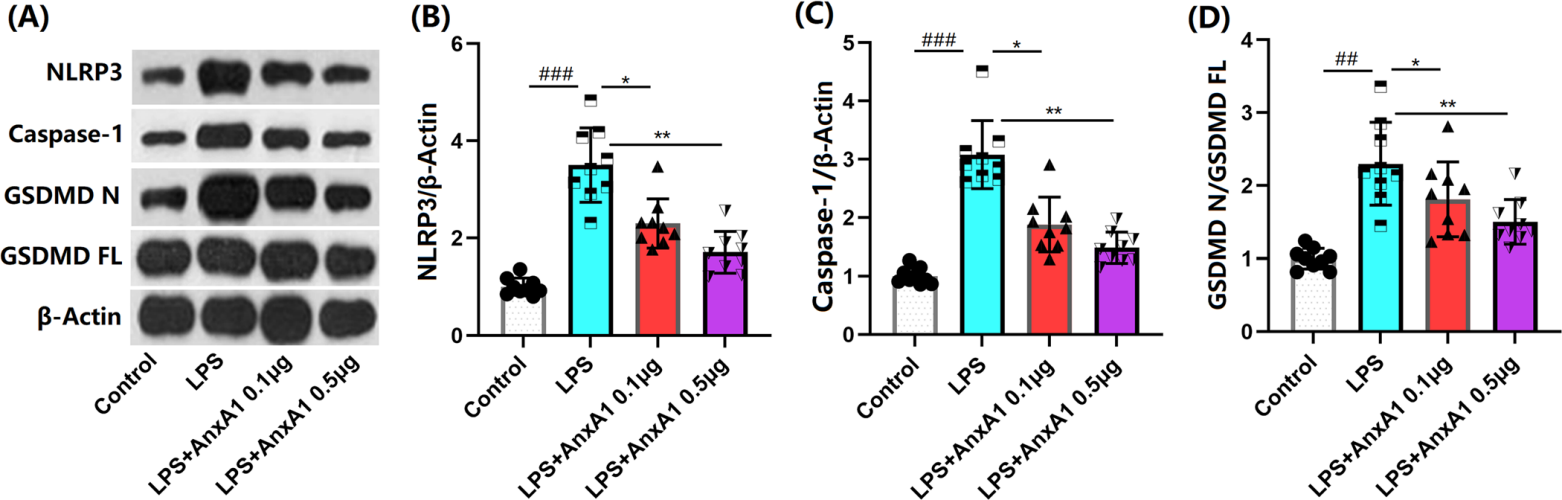
Annexin A1 suppressed the activation of NLRP3 signaling in the lung tissues of ALI mice. (A) The protein level of NLRP3, GSDMD N/GSDMD FL, and Caspase‐1 was determined by western blots; (B) the quantification of the blots (##, ###, *p* < 0.01, 0.005 vs. vehicle group; *, **, *p* < 0.05, 0.01 vs. ALI group, N = 9, two‐way ANOVA).

NLRP3 inflammasome activation is found to mediate the inflammation during ALI [18]. Herein, levels of NLRP3, Caspase‐1, and GSDMD N/GSDMD FL in lung tissues of ALI mice were significantly increased, which were remarkably repressed by 0.1 and 0.5 μg AnxA1, implying a repressive function of AnxA1 against NLRP3 activation during ALI (Figure 6A,B).

### 3.7 AnxA1 Activates Nrf2/HO‐1 Signaling in Lung Tissues of ALI Mice

**Figure 7:**
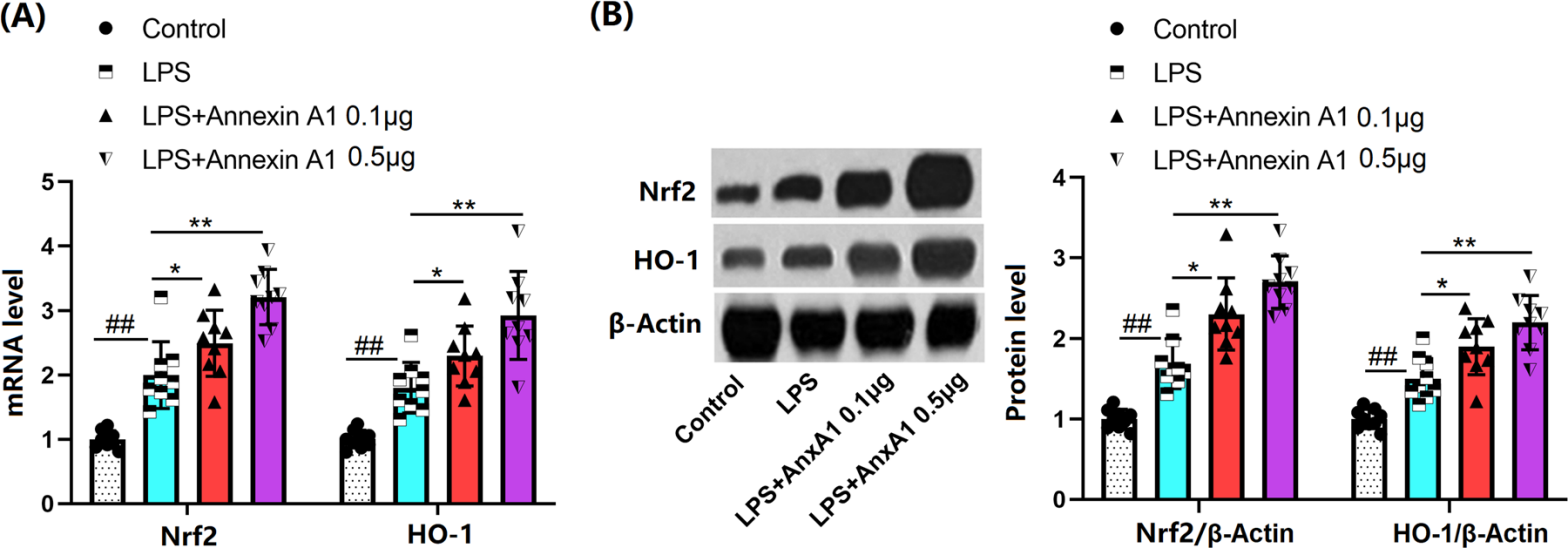
Annexin A1 activated the Nrf2/HO‐1 signaling pathway in the lung tissues of the acute lung injury (ALI) mouse model. (A) mRNA levels of Nrf2 and HO‐1; (B) protein levels of Nrf2 and HO‐1 in the lung tissues of ALI mice (##, *p* < 0.01 vs. vehicle group; *, **, *p* < 0.05, 0.01 vs. ALI group, *N* = 9, two‐way ANOVA).

The Nrf2/HO‐1 pathway is an anti‐OS signaling pathway involved in the progression of ALI [19]. Herein, levels of Nrf2 and HO‐1 in lung tissues were significantly enhanced in ALI mice, which were further strikingly boosted by 0.1 and 0.5 μg AnxA1, suggesting that AnxA1 activates the Nrf2/HO‐1 signaling during ALI (Figure 7).

### 3.8 AnxA1 Prevents NF‐κB Activation in ALI Mice

**Figure 8:**
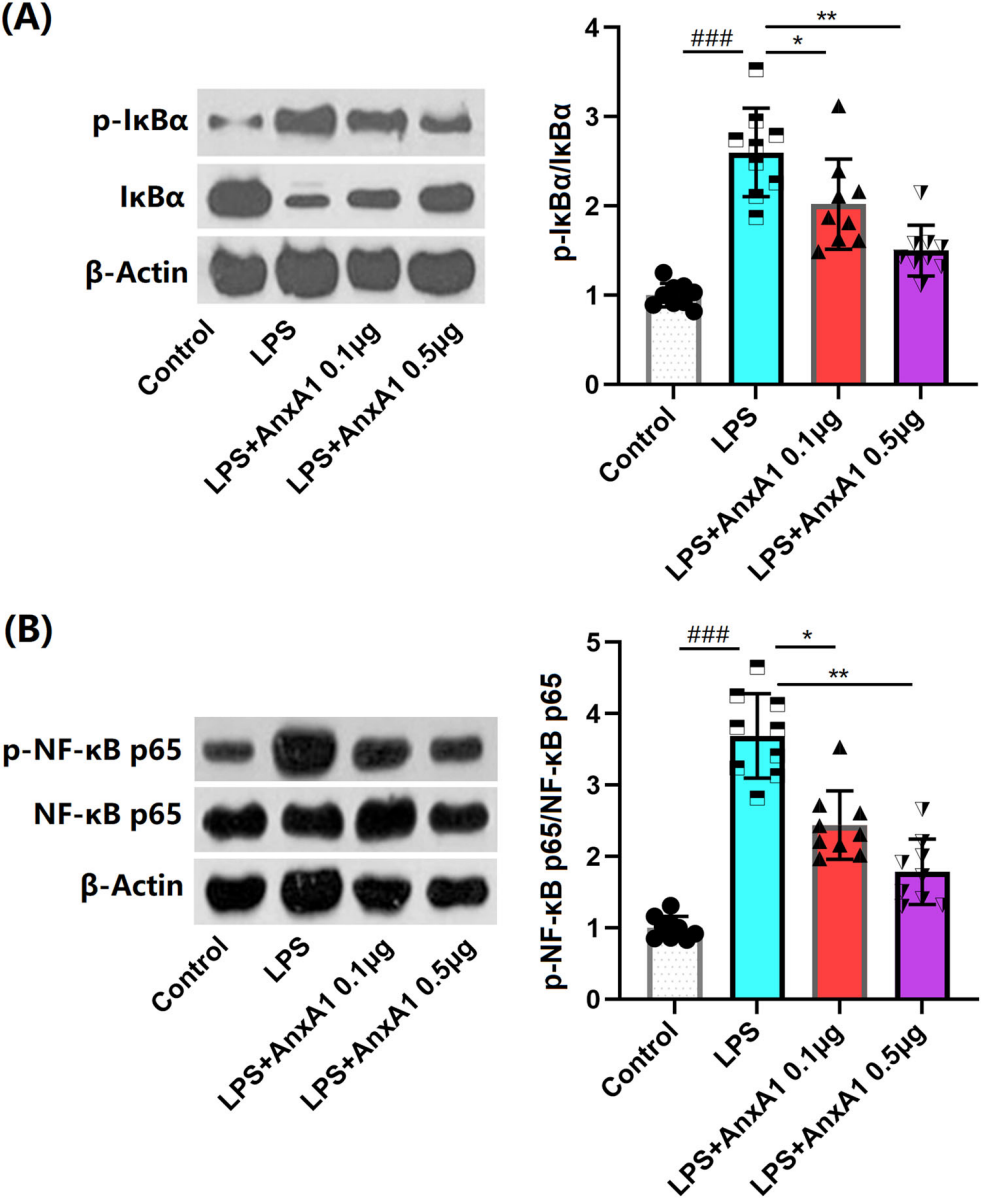
Annexin A1 prevented the activation of NF‐κB in the lung tissues of the ALI mouse model. (A) The levels of phosphorylated IκBα; (B) the levels of phosphorylated NF‐κB p65 (###, *p* < 0.005 vs. vehicle group; *, **, *p* < 0.05, 0.01 vs. ALI group, N = 9, two‐way ANOVA).

**Figure 9:**
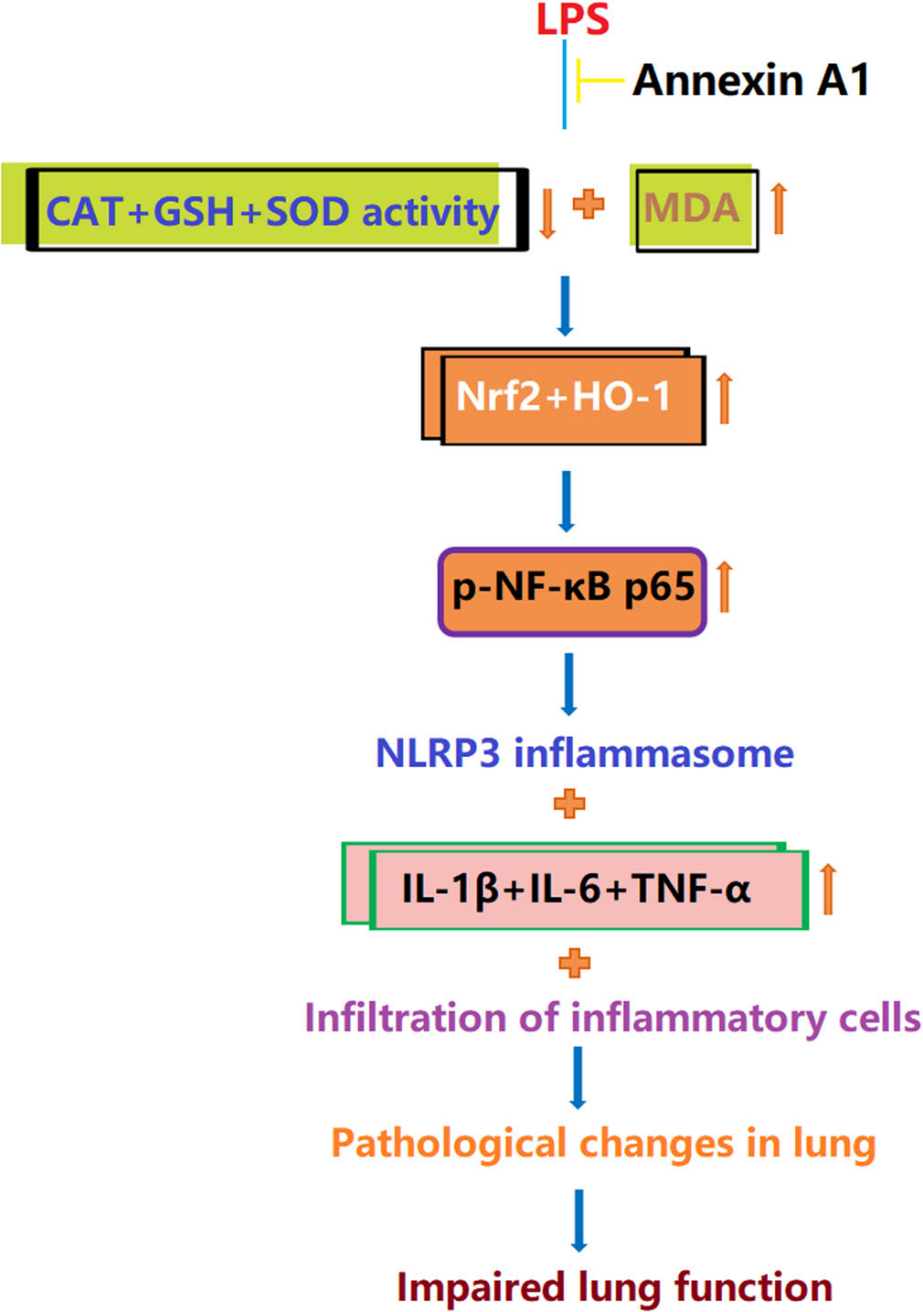
The graphic abstract of the study.

NF‐κB signaling is responsible for the generation of inflammatory cytokines during ALI [20]. Herein, levels of p‐IκBα (Figure 8A) and p‐NF‐κB p65 (Figure 8B) in lung tissues of ALI mice were considerably enhanced, which were markedly suppressed by 0.1 and 0.5 μg AnxA1. A graphic abstract is shown in Figure 9.

## 4 Discussion

Most scholars currently believe that excessive activation of inflammation in lung tissue or systemic inflammation caused by various factors is the primary pathogenesis of ALI [21, 22]. The cytokines involved in ALI include interleukins and tumor necrosis factors [23]. When lung inflammation is triggered, alveolar macrophages are the first responders, releasing various pro‐inflammatory cytokines. This activation of lung endothelial cells leads to the recruitment of mononuclear cells and neutrophils to the lung, triggering a cascade‐like release of cytokines. Consequently, this process further weakens the lung tissue's clearance ability and increases the permeability of lung microvessels. This process exacerbates lung inflammation, leading to progressive, refractory hypoxemia and gas exchange disorders, which can eventually result in acute respiratory distress syndrome (ARDS), respiratory failure, and patient death [24, 25]. This study aligns with Chen's report [26], which found that under LPS stimulation, severe pulmonary pathological changes and lung dysfunction were accompanied by enhanced inflammation in bronchoalveolar lavage fluid (BALF). The syndromes were significantly alleviated by AnxA1, indicating that AnxA1 inhibited the progression of ALI via the inhibition of inflammation.

The NLRP3 inflammasome is a multi‐component assembly comprising NOD‐like sensors, ASC, and Caspase‐1 [27]. During inflammatory conditions, the NLRP3 inflammasome is capable of recognizing danger signal molecules, such as LPS, both intracellularly and extracellularly. This recognition initiates a nonspecific immune response in the body, leading to the recruitment and activation of the proinflammatory protease Caspase‐1. Once activated, Caspase‐1 facilitates the maturation of IL‐1β. The activation of NLRP3 plays a crucial role in the progression of pulmonary pathological injury and is closely linked to the severity of ALI [28, 29]. As mentioned by Li [30] and Kang [31], NLRP3 signaling is significantly activated in ALI mice. However, this activation is notably suppressed by AnxA1. This suggests that AnxA1 might alleviate ALI by inhibiting NLRP3‐mediated inflammation. The NF‐κB pathway is a ubiquitous signaling mechanism within cells that participates in regulating various biological processes, including cell growth, differentiation, apoptosis, and inflammation [32]. NF‐κB is reported to play a role in the pathogenesis of ALI. When lung tissue is stimulated by various injurious factors, the NF‐κB pathway—a widely existing signaling mechanism—is activated. This activation regulates biological processes such as cell growth, differentiation, apoptosis, and inflammation. The triggered inflammatory response in pulmonary cells includes the release of inflammatory cytokines, chemokines, and coagulation factors. The release of these substances leads to extensive inflammatory reactions within lung tissue, further damaging both the tissue and its function [33]. By inhibiting the activation of NF‐κB, inflammatory reactions can be alleviated, thereby relieving the clinical symptoms of ALI [34]. In our study, consistent with previous reports [35, 36], NF‐κB activation was observed in ALI mice and was noticeably repressed by AnxA1. This indicates that AnxA1 might ameliorate ALI by inhibiting NF‐κB‐mediated inflammation.

OS refers to the excessive accumulation of oxygen free radicals and other ROS produced within cells. This accumulation leads to an imbalance in the cell's redox state, subsequently causing disruptions in cell structure and function [37]. In ALI, OS plays a crucial role in the pathophysiological process. Factors such as inhalation of harmful gases, pulmonary infections, and trauma can induce OS reactions in ALI. The excessive accumulation of oxygen free radicals and other ROS leads to lipid peroxidation, protein oxidation, DNA damage, and various oxidative stress responses in lung tissue. This results in the destruction of alveolar walls increased vascular permeability, exacerbated inflammation, and ultimately severe lung function impairment [7, 38]. In our study, consistent with Zeng's report [39], enhanced OS was observed in ALI mice, which was significantly ameliorated by AnxA1. This suggests that AnxA1 might alleviate ALI by suppressing OS. Heme oxygenase‐1 (HO‐1) is an important antioxidant enzyme, and its antioxidative function primarily relies on its byproducts carbon monoxide (CO) and biliverdin. Biliverdin and its reduced form, bilirubin, are effective antioxidants, while CO possesses anti‐inflammatory, antiapoptotic, and vasodilatory properties [40]. Under normal circumstances, the expression and activity of HO‐1 in the body are low. However, under stress conditions, such as exposure to LPS, ultraviolet radiation, hypoxia, etc., the expression of HO‐1 can be enhanced through Nrf2 to exert its antioxidative effects [41]. For example, in a mouse model of LPS‐induced ALI/ARDS, the Nrf2/HO‐1 pathway is activated and can alleviate ferroptosis to mitigate lung inflammation [17]. This indicates that the Nrf2/HO‐1 pathway is crucial for the body's antioxidative defense. In this study, consistent with data reported by Li [42], Nrf2/HO‐1 signaling in lung tissues of ALI mice was notably enhanced, and this enhancement was remarkably further activated by AnxA1. This suggests that AnxA1 might suppress OS by activating the Nrf2/HO‐1 pathway in ALI. Future studies will focus on exploring the specific downstream functional proteins of AnxA1 in regulating OS and inflammation to better illustrate the functional mechanism of AnxA1 in ALI.

There are several limitations to the current study. First, the molecular mechanism of AnxA1 in LPS‐induced ALI is not fully understood. While our findings suggest significant protective effects, further investigation is required to elucidate the detailed pathways through which AnxA1 acts. Specifically, it is important to understand how AnxA1 affects multiple cell types in the lung, and how epithelial cells and immune cells respond to AnxA1. Previous studies have shown that AnxA1 promotes neutrophil apoptosis and modulates apoptotic clearance by macrophages [43]. In experimental fibrosis animal models, AnxA1 protein expression increases in bronchial epithelial cells and leukocytes during the inflammatory phase [44]. Additionally, a recent study demonstrated that AnxA1 induces apoptosis in epithelial cells [45]. Therefore, the therapeutic effect of AnxA1 likely involves multiple cell types within lung tissue and may depend on cell‐cell interactions. Secondly, the current experiment can be further validated using AnxA1 null mice. This would allow us to attribute the effects of exogenous AnxA1 solely to its action in the absence of endogenous AnxA1. Thirdly, the physiological function of AnxA1 requires a more thorough investigation. While the full‐length AnxA1 was evaluated in the current study, the short form of AnxA1 peptide, an N‐terminal‐derived peptide of Annexin A1 (Ac2‐26), was also considered as an alternative. Further research is needed to fully understand the safety profile of both the full‐length AnxA1 and its peptide derivatives.

In summary, this study demonstrated that AnxA1 alleviated ALI in experimental mice by modulating inflammation and oxidative stress through regulating the NLRP3/NF‐κB and Nrf2/HO‐1 signaling pathways. Our findings suggest that AnxA1 may be a promising therapeutic agent for ALI.

## Author Contributions

**Hui Huang:** conceptualization; data curation; formal analysis; investigation; methodology; resources; visualization; writing–review & editing. **Yuqin Shi:** data curation; formal analysis; investigation; resources; validation; writing–review & editing. **Yuequan Zhou:** conceptualization; data curation; funding acquisition; project administration; software; supervision; visualization; writing–original draft.

## Ethics Statement

The experiments on animals were performed strictly in accordance with international animal welfare and ethical standards, as well as the relevant laws, regulations, and policies on the management of laboratory animals in Hubei province.

## Conflicts of Interest

The authors declare no conflicts of interest.

## Supporting information

Supporting information.

## Data Availability

The data supporting the findings of this study are available from the corresponding author upon reasonable request.
